# Soil mite communities (Acari: Mesostigmata) as indicators of urban ecosystems in Bucharest, Romania

**DOI:** 10.1038/s41598-021-83417-4

**Published:** 2021-02-15

**Authors:** M. Manu, R. I. Băncilă, C. C. Bîrsan, O. Mountford, M. Onete

**Affiliations:** 1 Department of Ecology, Taxonomy and Nature Conservation, Romanian Academy, Institute of Biology Bucharest, street Splaiul Independenţei, no. 296, PO-BOX 56-53, 0603100 Bucharest, Romania; 2 Faculty of Natural Sciences, University Ovidius Constanţa, Constanţa, Romania; 3 Department of Biospeleology and Soil Edaphobiology, “Emil Racoviţă” Institute of Speleology, Romanian Academy, 13 Septembrie Road, No. 13, 050711 Bucharest, Romania; 4 Centre for Ecology and Hydrology, Maclean Building, Benson Lane, Crowmarsh Gifford, Wallingford, OX10 8BB Oxfordshire UK

**Keywords:** Ecology, Zoology

## Abstract

The aim of the present study was to establish the effect of management type and of environmental variables on the structure, abundance and species richness of soil mites (Acari: Mesostigmata) in twelve urban green areas in Bucharest-Romania. Three categories of ecosystem based upon management type were investigated: protected area, managed (metropolitan, municipal and district parks) and unmanaged urban areas. The environmental variables which were analysed were: soil and air temperature, soil moisture and atmospheric humidity, soil pH and soil penetration resistance. In June 2017, 480 soil samples were taken, using MacFadyen soil core. The same number of measures was made for quantification of environmental variables. Considering these, we observed that soil temperature, air temperature, air humidity and soil penetration resistance differed significantly between all three types of managed urban green area. All investigated environmental variables, especially soil pH, were significantly related to community assemblage. Analysing the entire Mesostigmata community, 68 species were identified, with 790 individuals and 49 immatures. In order to highlight the response of the soil mite communities to the urban conditions, Shannon, dominance, equitability and soil maturity indices were quantified. With one exception (numerical abundance), these indices recorded higher values in unmanaged green areas compared to managed ecosystems. The same trend was observed between different types of managed green areas, with metropolitan parks having a richer acarological fauna than the municipal or district parks.

## Introduction

Over the past century, the world urban community has increased exponentially, with about half the world population living and working in urban areas that occupy only 2.8% of the land surface^1^. Urban areas contribute to human well-being from both economic and ecological points of view. Even if we discussed about the urban green ecosystems, each of them represent a dynamic complex of microorganism, plant and animal communities, which are correlated with abiotic factors (as: temperature, humidity of air and soil, acidity of soil, type of soil, etc.). In terms of ecosystem services, each type of ecosystems from natural to anthropic ones (as urban green areas) provides some benefits to people. Benefits, from ecological point of view, mean any services or goods that are used by the humans, directly or indirectly from nature. In general, urban areas, if well managed, can contribute to (or at least influence the delivery of) the following ecosystem goods and services: fresh water, food, timber, fiber, fuel, new products and industries from biodiversity; nutrient cycling, climate and air quality; ecosystem regulation of infectious diseases, waste processing and detoxification, regulation of natural hazards (floods and fires); and cultural services. In almost all cases, urban areas depend on nature. Human well-being depends on the health of urban and adjacent ecosystems^2–4^.

Biological diversity is a key component of almost all ecosystem services essential to human life. According to the Millennium Ecosystem Assessment, biodiversity contributes to human security, resilience, health and freedom of choice and action^5,6^. In general, cities are characterised by the following features: high density of human population, increased numbers of industrial, business and residential areas, high anthropogenic impact on natural habitats, higher temperatures in comparison to adjacent ecosystems, extinction of native species and an increased impact and number of invasive and allochthonous plant and animal species^6,7^. All these factors, together with increased urbanisation, lead to high pressure on ecosystems and biodiversity within the urban environment.

Soil invertebrates are a valuable component of biodiversity. They influence nutrient cycling by feeding directly on plant materials and organic substrates. They have a direct or indirect influence on litter decomposition, having a consistent positive effect on this soil process at global and biome scales^8,9^. Some components of the soil fauna are ecosystem engineers, influencing soil structure, as well as mineral and organic matter composition^10^. Mites (Acari) are one of the most abundant groups of soil invertebrate. Up to the present day, approximately 55,000 species of mite have been recognised and described, although researchers have estimated that the actual total may be between 500,000 and 1,000,000^11^. Research has highlighted several important reasons for studying invertebrates in urban areas: (a) they represent a good assessment tool for biodiversity status; (b) they respond rapidly to any environmental disturbance (due to their short generation times); (c) they are easy to collect; (d) they are present at many trophic levels; and (e) are important in terms of ecosystem services correlated with anthropogenic changes^12,13^.

Predatory mites (Acari-Mesostigmata) are free-living species which occupy various niches that are primarily or secondarily related to soil and litter (e.g. litter-fermentation layers, moss, bark, dead wood, trunks, stumps, tree-hollows and nests) present within different types of ecosystems e.g. forest, scrub, dunes, grassland, agricultural land and urban areas^14–23^. Together with other small soil invertebrates (springtails, enchytraeids, insect larva, oribatids), they participate indirectly in the decomposition process and ultimately affect soil quality (fertility and productivity)^4,10^. At the same time, they are the main regulator of the other soil invertebrate populations^11,24,25^. Most species prefer habitats rich in organic matter, with high soil moisture, medium soil temperature and a low pH^26–28^. Because of these ecological requirements and their marked sensitivity to environmental/anthropogenic disturbance, soil mites are often used as soil bioindicators^15,29–33^.

Research studies on predatory soil mites (Acari-Mesostigmata) from cities around the world are relatively few, either in urban sites (forests, roadsides, greeneries, gardens, galleries, parks, housing estates, town centres, grasslands, cemeteries, botanical gardens) or in suburban areas and industrial areas. Such studies have been conducted in Europe (Latvia, Poland, Italy, Slovakia, Hungary) or in the USA, but they focussed on all soil-invertebrate communities, on Uropodina, as a suborder of Mesostigmata order or only on few urban habitats^29,34–45^. Some of these studies investigated phoretic species, especially those from the Uropodina suborder^46–48^. In Romania, there are few studies of the Mesostigmata fauna in urban areas, all located in three Bucharest parks and two forests close to the city^21–23,32,49–51^. All these European and national studies are mainly faunistic or taxonomic, almost without any information on the ecological aspect of soil mite communities in relation to urban environmental factors.

To address this research gap, we focussed on the communities of Mesostigmata soil mites in managed and unmanaged urban areas in Bucharest, through a more extensive study. The project also includes one of the few urban protected areas in Europe (Văcăreşti Natural Park). The main objectives of the study were investigated: (1) the effect of management regime in urban areas on the community structure, abundance and species richness of mesostigmatid mites; (2) some key urban environmental variables; and (3) the influence of the selected abiotic parameters on the structure of mesostigmatid communities in the ecosystems studied (as: soil and air temperature, soil moisture and atmospheric humidity, soil pH and soil penetration resistance).

## Material and methods

### Study area

**Figure 1:**
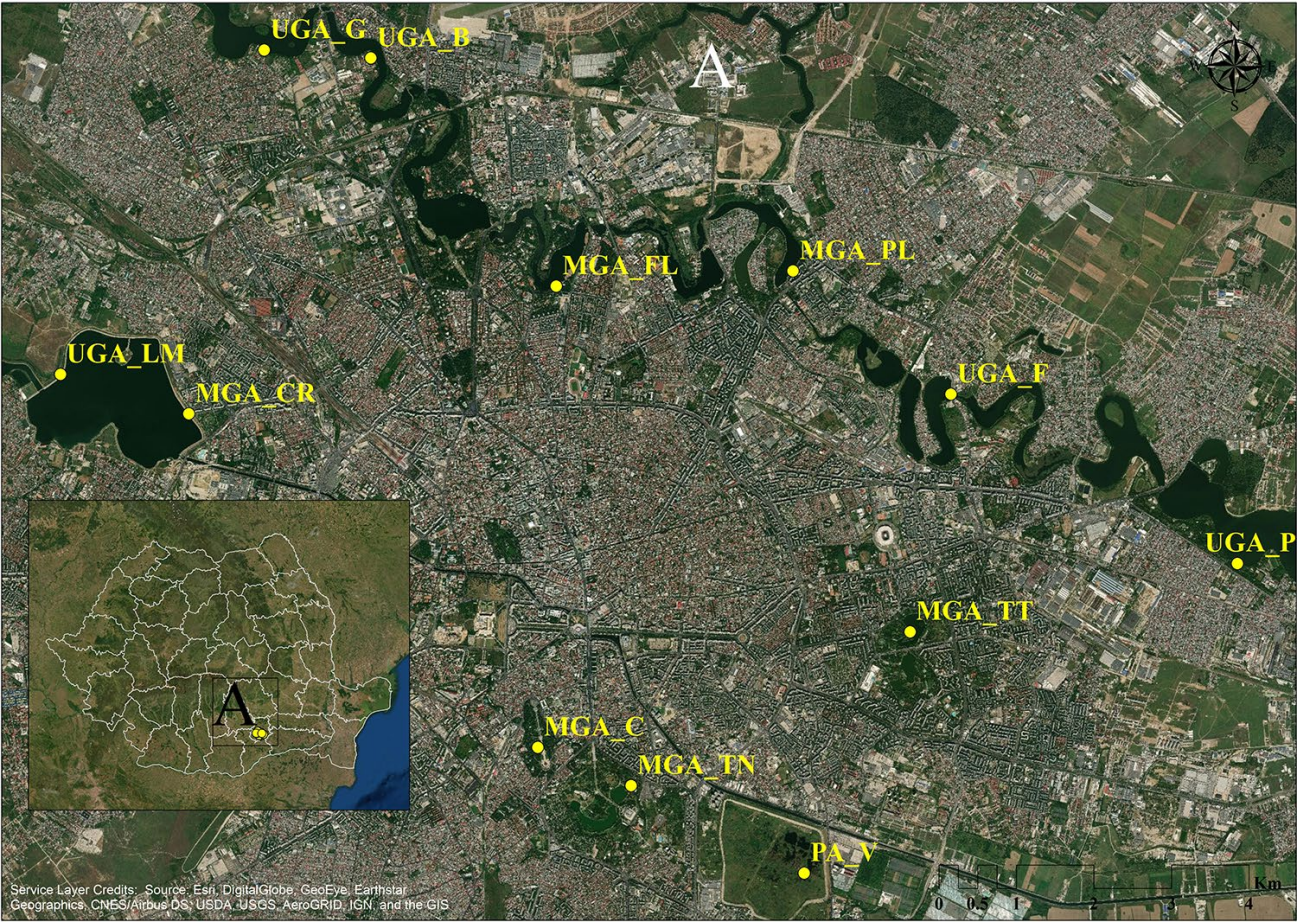
Geographical locations of the natural protected urban area (PA), managed (MGA) and unmanaged green areas (UGA) in Bucharest, investigated in 2017. Map created using ArcGIS software by Esri. ArcGIS and ArcMap are the intellectual property of Esri and are used herein under license. Copyright Esri. All rights reserved. For more information about Esri software, please visit www.esri.com.

The study was conducted in June 2017, in twelve urban green areas in Bucharest (Fig. 1). Bucharest is situated in Central Eastern Europe, in the lower Danube region. The mean annual temperature is about 10–11 °C, and annual precipitation is 615 mm^52^. Of the total city area (22,800 ha), urban green space occupies 2275 ha (9.97%) while urban parks represent 29.9% of Bucharest and approximately 3% of the total city area but are unequally distributed. Urban parks were classified as (a) metropolitan: MtP (average area = 52.4 ha, over 5000 visitors/weekend day), (b) municipal: MnP (average area = 40.3 ha, 2000–5000 visitors/weekend day), and (c) district: DrP (average area = 6.4 ha, under 2000 visitors/weekend day)^52,53^.

The ecosystems studied were classified in three management scenarios (MS): (1) urban protected area (PA) i.e. Văcăreşti (V), (2) managed green urban areas (MGA) i.e. metropolitan parks [Tineretului (TN) and Titan (TT)], municipal parks (Plumbuita (PL) and Carol (C) and district parks (Floreasca (FL) and Crângaşi (CR), and (3) unmanaged green urban areas (UGA) i.e. Băneasa (B), Pantelimon (P), Griviţa (G), Lacul Morii (LM) and Fundeni (F). A detailed description of these areas is given in Supplementary Appendix 1. Văcăreşti Natural Park is the biggest (183 ha) and the newest park designated in Bucharest as well as the first urban nature park in Romania. It is located at 5 km distance from the city centre and it was classified in the category V (“protected landscape”) following the IUCN criteria^54^.

In all managed green areas (with one exception—Văcăreşti Natural Park), the local administrations together with the Plant Protection Centre subordinated to the General Council of Bucharest carries out treatments to combat plant pests (through biological and integrated methods): mites, aphids, thrips, moths, powdery mildew, rust, wasps, defoliating caterpillars, San Jose lice, woolly lice, cicadas, etc. Weed control is also applied in all managed green areas, using manual methods. These methods are governed by the Regulation of the public service for the administration of the public and private domain, regarding the activities of arrangement and maintenance of the parks and of the gardens in the municipality of Bucharest. All managed areas are irrigated.

### Soil fauna

Soil samples were collected in June 2017, using a MacFadyen soil core (5 cm diameter) to 10 cm depth. In total, 480 soil samples were collected randomly (40 samples/urban area). In each urban ecosystem, the sampled area was 100 m^2^ Due to the small number of immature individuals, they were not considering separately in our statistical analysis. The mites were extracted with a Berlese-Tullgren funnel, in ethyl alcohol, clarified in lactic acid and identified to species level, using published identification keys^55–63^. The mites extraction lasted 10 days. Some specimens were mounted on permanent slides. All species were deposited in the collection of the Institute of Biology-Bucharest, Romanian Academy-Research Station Posada.

### Environmental variables

In order to establish the relationship between soil mite communities and environmental variables, we measured air and soil temperature (T-air, T-soil as °C), air and soil moisture (H-air and H-soil as %), soil pH, and soil penetration resistance (RP as MPa). Air and soil moisture and temperature were measured with a digital thermo-hygrometer PCE-310. Air temperature and moisture were measure at 5 cm above the soil level. Penetration resistance was determined with a soil penetrometer, Step System GmbH, 41,010. The pH was measured with a C532 Jasco Consort pH-meter. The average values of environmental variables are presented in Tables 1 and 2.

**Table 1:** Mean ± standard deviation (range) of the environmental variables for the different categories of management regime in urban areas.

Environmental variable	MGA	PA	UGA	Total	p value
T soil (°C)	16.34 ± 2.80 (10.10–25.60)	16.87 ± 1.52 (15.20–20.50)	14.5 ± 2.77 (9.80–23.20)	15.51 ± 2.86 (9.80–25.60)	< 0.001
T air (°C)	20.51 ± 4.26 (10.40–28.90)	30.02 ± 1.78 (26.50–32.80)	26.30 ± 3.91 (18.20–32.50)	24.08 ± 5.14 (10.40–32.80)	< 0.001
H soil (%)	13.16 ± 6.94 (1.03–30.00)	12.56 ± 8.07 (0.81–33.80)	11.95 ± 4.72 (1.73–25.50)	12.53 ± 6.09 (0.81–33.80)	0.334
H air (%)	75.4 ± 17.37 (44.00–92.00)	54.95 ± 7.27 (37.00–69.00)	54.39 ± 6.69 (40.00–68.00)	63.76 ± 16.37 (37.00–92.00)	< 0.001
pH	8.24 ± 0.26 (7.64–8.84)	8.40 ± 0.25 (7.99–8.95)	8.35 ± 0.51 (7.35–9.67)	8.31 ± 0.40 (7.35–9.67)	0.061
RP (Mpa)	1.8 (0.59 ± 0.68–2.75)	1.63 ± 0.53 (0.82–2.55)	1.16 ± 0.40 (0.34–2.06)	1.48 ± 0.59 (0.34–2.75)	< 0.001

The p value represents the significance level of the global one-way analysis of variance performed separately for each environmental variable.

**Table 2:** Mean ± standard deviation (range) of the environmental variables for the different categories of managed green areas.

Environmental variable	MtP	MnP	DrP	Total	p value
T soil (°C)	18.06 ± 2.15 (15.80–25.60)	17.08 ± 0.95 (15.70–19.50)	13.41 ± 2.148 (10.10–17.20)	16.34 ± 2.80 (10.10–25.60)	< 0.001
T air (°C)	21.60 ± 4.83 (16.20–28.90)	17.94 ± 1.05 (16.20–19.30)	20.95 ± 4.23 (10.40–26.00)	20.51 ± 4.26 (10.40–28.90)	0.001
H soil (%)	15.63 ± 8.00 (1.03–30.00)	11.74 ± 4.26 (4.52–20.80)	10.84 ± 5.98 (3.10–25.00)	13.16 ± 6.94 (1.03–30.00)	0.004
H air (%)	71.64 ± 21.2 (44.00–92.00)	90.65 ± 0.69 (89.00–91.00)	69.68 ± 10.29 (54.00–85.00)	75.64 ± 17.38 (44.00–92.00)	< 0.001
pH	8.16 ± 0.23 (7.64–8.64)	8.25 ± 0.3 (7.67–8.65)	8.35 ± 0.24 (7.98–8.84)	8.24 ± 0.26 (7.64–8.84)	0.006
RP (Mpa)	1.78 ± 0.53 (0.68–2.75)	2.11 ± 0.54 (0.68–2.75)	1.59 ± 0.60 (1.03–2.75)	1.79 ± 0.59 (0.68–2.75)	0.002

The p value represents the significance level of the global one-way analysis of variance performed separately for each variable.

### Data analysis

The completeness of the species inventory was examined by visual inspection of Mao Tau species accumulation curves for each management scenario and for pooled data of all sites.

We examined differences across management scenarios: managed green areas, unmanaged green areas and sites (Băneasa, Pantelimon, Griviţa, Lacul Morii, Fundeni, Carol, Plumbuita, Tineretului, Titan, Floreasca and Crângaşi) using non-metric multidimensional scaling (NMDS) with the Bray–Curtis (B–C) distance index.

The NMDS was conducted using the function “metaMDS” in the R package “*vegan*”^64^^.^ The function automatically applies a square root transformation to the data matrix of species abundance. The environmental variables were standardised to have mean zero and unit variance. We ran NMDS using 500 random starts and tested the goodness of fit of the data using the R2 value and examining the Shepard plot (i.e. the scatter around the regression of the distances between each pair of communities against their original dissimilarities). The significance of differences between communities from management scenarios and sites was assessed with an overall PERMANOVA based on B–C dissimilarities with the function “adonis” within the *vegan* package and pairwise using the function “pairwise.perm.manova” within the “RVAideMemoire” package^65^. The same analyses were repeated to examine the differences among different types of MGAs, i.e. metropolitan, municipal and district parks.

To determine whether physical characteristics influenced the species communities, we conducted a canonical correspondence analysis (CCA). Before performing the CCA, we selected the significant environmental variables using the ANOVA permutation test for CCA (999 random permutation) and only introduced the variables with P > 0.05 in the CCA.

We used linear mixed models (LMMs) to test whether the main community feature (i.e. the species richness SR) was related to: (i) management scenario, (ii) environmental variables and (iii) a combination of management scenarios and environmental variables. In the LMMs, the management scenario and the environmental variables were introduced as fixed effects and sites as random effects. We assessed the relative performance of the models using the selection technique based on Akaike’s information criterion corrected for sample size—AICc^66,67^. We ranked the models and the model with the lowest AICc was used as the reference for calculating the AIC difference (Δi) and the likelihood of a model given the data and model weights (wi). Models within two AIC units of the AICmin were considered competitive and more plausible than others^66^.

To identify any taxonomic group-specific pattern across management scenarios we examined the extent to which each management scenario was characterised by rare species, i.e. singletons and doubletons. All analyses were performed using R version 3.2.1.

Based on the reproduction strategy of soil mites (“k” or “R”)^68^, the index of maturity was calculated for each studied urban green area (Supplementary Appendix 2). Using PAST software, we quantified the following parameters: dominance (D), Shannon index of diversity (H) and equitability (J)^69^.

To identify mite species characteristic to the three MS we applied the indicator Value method using the “indicspecies” R package^70,71^. The method assesses the specificity (uniqueness to a particular MS) and fidelity (frequency of occurrence) of mite species. A high indicator value (IndVal, expressed as percentage) indicates that a mite species can be considered characteristic to a particular or combination of MS. The IndVal for each mite species was calculated based on the matrix of mite species abundance. We used a random reallocation procedure of sites among site groups to test for the significance of IndVal measures for each mite species^70^. According to Dufrêne and Legendre (1997), a mite species is considered characteristic to a MS or combination of MS if the species IndVal is > 25% and significant at p < 0.05.

## Results

**Figure 2:**
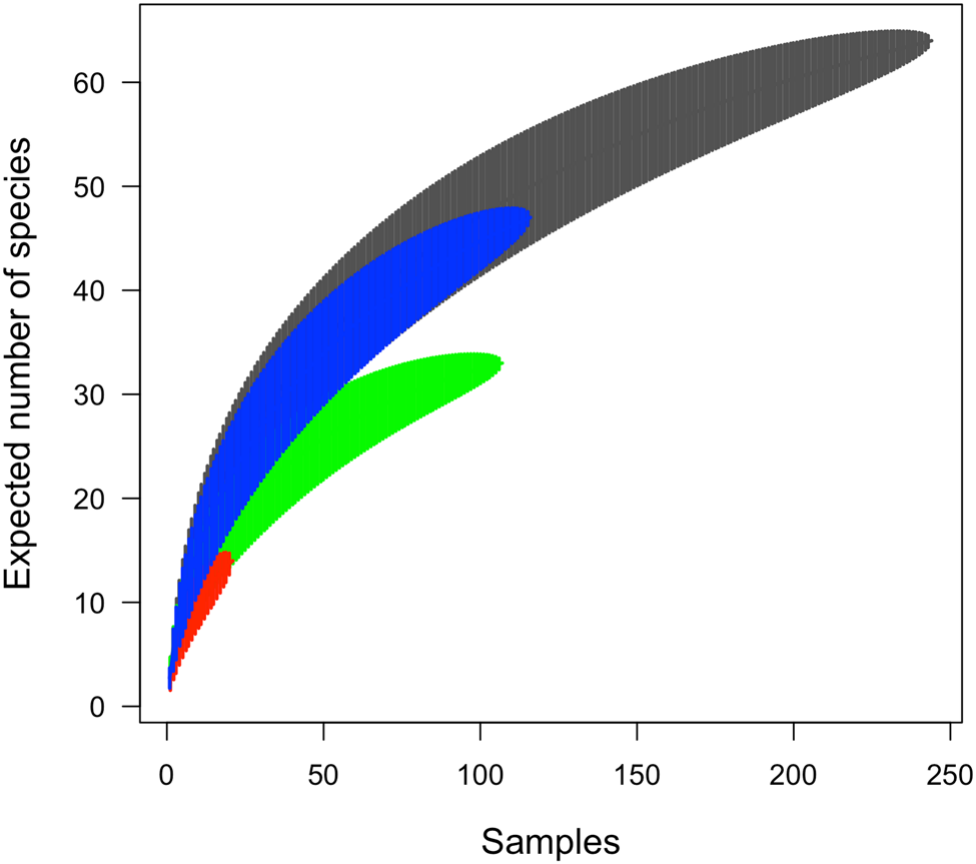
Mao Tau species accumulation curves per management scenarios: (**a**) protected areas (PA)-red colour, (**b**) managed green areas (MGA)-green colour, (**c**) unmanaged green areas (UGA)—blue colour, and (**d**) for all sites—grey colour.

Analysing the entire Mesostigmata community, we discovered 68 species, with 790 individuals and 49 immatures. If we consider the protected area, 8 species were identified, with 37 individuals and 15 immatures. In managed urban areas 33 species were identified, with 435 individuals and 25 immatures while in unmanaged green ecosystems, we recorded 49 species, with 318 individuals and 9 immatures (Tables 3, 4). None of the species accumulation curves for each management scenario or for pooled data from all sites approached an asymptote, indicating that more samples are required to detect all the species theoretically expected (Fig. 2).

**Table 3:** Numerical abundance of Mesostigmata mites from MGA areas (including the urban protected area-V) in Bucharest, 2017.

No	Species	Code	V	TN	TT	C	PL	FL	CR	Total MGA	Total MGA + PA
	**Family Parasitidae**										
1	*Lysigamaus* sp.	*Ly.sp*	0	0	0	0	0	0	4	4	4
2	*Pergamasus laetus* Juvara-Bals, 1970	*Pe.la*	0	0	0	0	0	0	3	3	3
3	*Pergamasus quisquiliarum* (R. and G. Canestrini, 1882)	*Pe.qu*	0	11	17	8	2	0	2	40	40
4	*Pergamasus crassipes* (Linnaeus, 1758)	*Pe.cr*	2	1	1	0	0	0	5	7	9
5	*Paragamasus similis* (Willmann, 1953)	*Pa.si*	0	0	0	1	0	0	0	1	1
6	*Parasitus beta* (Oudemans et Voigts, 1904)	*Pa.be*	0	0	0	0	0	0	4	4	4
7	*Parasitus fimetorum* (Berlese, 1904)	Pa.fi	1	0	0	0	0	0	0	0	1
8	*Parasitus insignis* (Holzmann, 1969)	*Pa.in*	0	0	1	0	0	1	0	2	2
9	*Vulgarogamasus kraepelini* (Berlese, 1905)	*Vu.kr*	0	0	0	0	2	1	0	3	3
	**Family Veigaiidae**										
10	*Veigaia exigua (Berlese, 1916)*	*Ve.ex*	0	4	0	0	0	0	2	6	6
11	*Veigaia nemorensis* (C.L.Koch, 1836)	*Ve.ne*	0	1	0	0	1	0	3	5	5
12	*Veigaia planicola* (Berlese, 1892)	*Ve.pl*	2	15	2	8	0	3	0	18	30
	**Family Digamasellidae**										
13	*Dendroelalaps foveolatus* (Leitner, 1949)	*De.fo*	0	53	1	1	0	0	0	55	55
	**Family Rhodacaridae**										
14	*Rhodacarellus silesiacus* Willmann, 1936	*Rh.si*	9	2	7	6	0	3	19	37	46
15	*Rhodacarus denticulatus* (Berlese, 1921)	*Rh.de*	1	0	0	0	5	2	0	7	8
	**Family Ascidae**										
16	*Arctoseius cetratus* (Sellnick, 1940)	*Ar.ce*	0	12	17	1	1	0	0	31	31
17	*Cheroseius bryophilus* Karg, 1969	*Ch.br*	0	0	2	0	0	0	0	2	2
18	*Gamasellodes bicolor* (Berlese, 1918)	*Ga.bi*	0	5	0	0	10	1	0	16	16
19	*Proctolaelaps pygmaeus* (Muller, 1859)	*Pr.py*	0	1	0	0	0	0	4	5	5
20	*Protogamasellus singularis* (Karg, 1962)	*Pr.si*	0	0	0	0	3	0	0	3	3
21	*Leioseius minusculus* Berlese, 1905	*Le.mi*	0	0	1	0	0	0	0	1	1
	**Family Halolaelapidae**										
22	*Halolaelaps* sp.	*Ha.sp*	0	0	0	0	2	0	0	2	2
	**Family Ameroseiidae**										
23	*Ameroseius* sp.	*Am.sp*	0	0	1	0	0	0	0	1	1
	**Family Pachylaelapidae**										
24	*Olopachys vysotskajae* Koreleva, 1976	*Ol.vy*	0	0	0	2	1	0	0	3	3
25	*Onchodellus karawaiewi* (Berlese, 1920)	*On.ka*	3	7	0	10	0	0	0	17	20
	**Family Laelapidae**										
26	*Hypoaspis aculeifer* Berlese 1892	*Hy.ac*	14	32	52	13	16	9	7	129	143
27	*Hypoaspis praesternalis* Willmann, 1949	*Hy.pr*	5	9	0	0	0	0	3	12	17
28	*Hypoaspis vacua* (Michael, 1891)	*Hy.va*	0	1	0	0	0	2	0	3	3
29	*Pseudolaelaps doderoi* (Berlese, 1910)	*Ps.do*	0	0	0	0	0	1	0	1	1
30	*Laelaps astronomica* Koch, 1839	*La.as*	0	0	0	0	0	2	0	2	2
	**Family Nenteriidae**										
31	*Nenteria* sp.	*Ne.sp*	0	1	0	0	0	0	0	1	1
	**Family Uropodidae**										
32	*Pseudouropoda* sp.	*Ps.sp*	0	0	2	0	0	0	0	2	2
33	*Uropoda* sp.	*Uro.sp*	0	1	0	0	0	0	0	1	1
	**Family Urodinychidae**										
34	*Urodiaspis* sp.	*Ur.sp*	0	1	0	0	0	0	0	1	1
	Total no. of species		8	17	12	9	10	10	11	33	34
	Total no. of individuals		37	157	104	50	43	25	56	435	472
	Total no. of immatures		15	3	6	1	6	9	0	25	40
	Index of maturity		0.61	0.50	0.34	0.66	0.37	0.33	0.58		
	Dominance_D		0.234	0.189	0.310	0.176	0.219	0.184	0.165		
	Shannon_H		1.696	2.085	1.572	1.876	1.834	1.998	2.116		
	Equitability_J		0.816	0.736	0.632	0.854	0.796	0.868	0.883

**Table 4:** The numerical abundance of Mesostigmata mites from UGA areas from Bucharest, 2017.

No	Species	Code	B	P	G	LM	F	Total
	**Family Parasitidae**							
1	Lysigamasus sp.	Ly.sp	0	3	0	0	1	4
2	*Pergamasus crassipes* (Linnaeus, 1758)	Pe.cr	1	2	0	0	7	10
3	*Parasitus beta* (Oudemans et Voigts, 1904)	Pa.be	0	0	1	0	0	1
4	*Parasitus fimetorum* (Berlese, 1904)	Pa.fi	1	0	4	1	0	6
5	*Parasitus insignis* (Holzmann, 1969)	Pa.in	2	0	0	0	0	2
6	*Vulgarogamasus kraepelini* (Berlese, 1905)	Vu. kr	0	1	0	0	0	1
7	*Holoparasitus calcaratus* (CL Koch, 1839)	Ho.ca	0	0	4	0	0	4
	**Family Ologamasidae**							
8	Euryparasitus sp.	Eu.sp	0	0	1	0	0	1
9	Sessiluncus sp.	Se. sp	0	0	2	0	0	2
	**Family Veigaiidae**							
10	*Veigaia exigua* (Berlese, 1916)	Ve.ex	0	4	2	0	6	12
11	*Veigaia nemorensis* (C.L.Koch, 1836)	Ve.ne	0	1	1	0	6	8
12	*Veigaia planicola* (Berlese, 1892)	Ve.pl	7	37	6	1	35	86
13	Veigaia sp.	Ve.sp.	0	0	0	0	1	1
	**Family Digamasellidae**							
14	Dendrolaelaps sp.	De.sp.	0	0	0	1	16	17
	**Family Rhodacaridae**							
15	*Rhodacarellus silesiacus* Willmann, 1936	Rh.si	0	8	9	11	5	33
16	*Rhodacarus denticulatus* (Berlese, 1921)	Rh.de	3	0	0	0	0	3
17	*Rhodacarus roseus* Oudemans, 1902	Rh.ro	1	0	0	0	0	1
	**Family Ascidae**							
18	*Arctoseius semiscissus* Berlese, 1892	Ar.se	0	0	0	0	1	1
19	*Asca bicornis* (Canestrini and Fanzago, 1887)	As.bi	2	0	0	0	0	2
20	*Cheiroseius curtipes* (Halbert, 1923)	Ch.cu	0	0	0	0	1	1
21	Lasioseius sp.	Las.sp	0	0	0	1	0	1
	**Family Ameroseiidae**							
22	Ameroseius sp.	Am.sp	0	0	1	1	0	2
	**Family Macrochelidae**							
23	*Geholaspis mandibularis* (Berlese 1904)	Ge.ma	1	0	1	0	0	2
24	*Glyptholaspis americana* (Berlese, 1888)	Gl.am	0	0	0	1	0	1
25	*Macrocheles recki* Bregetova and Koroleva, 1960	Ma.re	3	1	3	0	0	7
26	Macrocheles sp.	Ma.sp	1	0	1	0	0	2
	**Family Pachylaelapidae**							
27	*Olopachys suecicus* Sellnick, 1950	Olo.sp	0	1	0	0	0	1
28	*Olopachys vysotskajae* Koreleva, 1976	Ol.vy	0	0	2	0	0	2
29	*Onchodellus karawaiewi* (Berlese, 1920)	On.ka	0	4	2	1	0	7
30	Onchodellus sp.	On.sp.	2	0	0	0	0	2
31	*Pachydellus furcifer* (Oudemans 1903)	Pa.fu	0	2	0	0	2	4
32	Pachyseius sp.	Pa.sp	2	0	1	0	0	3
33	*Pachylaelaps pectinifer* (G. et R. Canestrini, 1882)	Pa.pe	0	0	0	0	1	1
	**Family Laelapidae**							
34	*Hypoaspis aculeifer* Berlese 1892	Hy.ac	8	9	4	13	12	46
35	*Hypoaspis claviger* (Berlese,1883)	Hy.cl	0	0	2	0	0	2
36	*Hypoaspis karawaiewi* (Berlese, 1904)	Hy.ka	0	0	0	1	0	1
37	*Hypoaspis praesternalis* Willmann, 1949	Hy.pr	0	0	0	0	1	1
38	Hypoaspis sp.	Hy.sp.2	1	0	0	0	0	1
39	*Hypoaspis vacua* (Michael, 1891)	Hy.va	1	0	0	0	1	2
40	Laelaps sp.	La.sp	2	0	0	1	0	3
41	*Pseudolaelaps doderoi* (Berlese, 1910)	Ps.do	0	0	2	0	0	2
	**Family Eviphididae**							
42	*Alliphis halleri* (G and C Canestrini, 1881)	Al.ha	5	0	0	4	10	19
	**Family Zerconidae**							
43	*Zercon fageticola* Halaskova, 1970	Ze.fa	1	0	0	0	0	1
	**Family Trachytidae**							
44	*Trachytes baloghi* Hirschmann and Zirngiebl-Nicol, 1969	Tr.ba	1	0	0	0	0	1
45	*Trachytes pauperior* Berlese, 1914	Tr.pa	1	0	0	0	0	1
	**Family Nenteriidae**							
46	Nenteria sp.	Ne.sp	1	0	0	0	0	1
	**Family Urodinychidae**							
47	Dinychus sp.	Di.sp	0	0	0	1	0	1
	**Family Uropodidae**							
48	Uropoda sp.	Uro.sp	1	0	0	0	3	4
49	Olodiscus sp.	Ol.sp	0	0	0	0	1	1
	Total no. of species		22	12	19	13	18	49
	Total no. of individuals		48	73	49	38	110	318
	Total no. of immatures		1	0	2	0	6	9
	Index of maturity		0.40	0.71	0.6	0.29	0.45	
	Dominance_D		0.082	0.294	0.085	0.218	0.156	
	Shannon_H		2.791	1.726	2.692	1.92	2.251	
	Equitability_J		0.903	0.695	0.914	0.749	0.779

Examining the mean values for environmental variables, we observed that soil temperature, air temperature, air humidity and soil penetration resistance differed significantly between all three types of managed urban green area (p < 0.05). The MGA areas were characterised by the highest mean values of air and soil moisture, RP and by the lowest mean values of air temperature and pH. The PA had the highest mean values of air and soil temperature, pH and moderate mean values of the remaining abiotic factors. In UGA, the lowest mean values of soil temperature, RP air and soil moisture were recorded (Table 1).

Measuring the environmental variables from the three types of MGA, we recorded a significant difference between their mean values (p < 0.05). MtP was characterised by the highest mean values of air and soil temperatures, soil moisture and the lowest mean value of pH. MnP were characterised by the highest values of atmospheric humidity (RP) and lowest air temperature. In DrP, the lowest average values of soil temperature and moisture (RP) and the highest mean value of pH were observed (Table 2).

The Shepard plot showed that original dissimilarities were well preserved in the reduced number of dimensions and low stress was found for both NMDS analyses examining the differences across the MS (R2 = 1, stress = 0.001) and different types of MGA (R2 = 0.966, stress = 0.055) (Supplementary Appendix 3), respectively.

The NMDS analysis showed low clustering of data points by both management scenarios, types of MGA and sites (Supplementary Appendix 4).

PERMANOVA showed significant differences between MS (F_[2,243]_ = 3.77, R2 = 0.03, p < 0.001), MGA (F_[2,106]_ = 2.91, R2 = 0.050 = 3, p < 0.001) and sites (F_[11,243]_ = 3.04, R2 = 0.126, p < 0.001). Pairwise comparisons of species communities identified marginally significant differences only between MGA and UGA (p = 0.05) and DrP and MtP (p = 0.05). Comparisons between species assemblages between all pairs of sites are shown in Table 5.

**Table 5:** Results of MANOVA pairwise comparisons of species communities among sites: Baneasa (B), Pantelimon (P), Grivita (G), Lacul Morii (LM), Fundeni (F), Carol (C), Plumbuita (PL), Tineretului (TN), Titan (TT), Floreasca (FL), Cringasi (CR), Văcăreşti (V).

	B	C	CR	F	FL	G	LM	P	PL	TN	TT
C	0.211	–	–	–	–	–	–	–	–	–	–
CR	0.049	0.049	–	–	–	–	–	–	–	–	–
F	0.115	0.073	0.049	–	–	–	–	–	–	–	–
FL	0.418	0.364	0.092	0.162	–	–	–	–	–	–	–
G	0.073	0.073	0.092	0.049	0.115	–	–	–	–	–	–
LM	0.049	0.073	0.092	0.049	0.330	0.073	–	–	–	–	–
P	0.049	0.049	0.049	0.318	0.073	0.049	0.049	–	–	–	–
PL	0.44	0.092	0.049	0.115	0.138	0.049	0.049	0.049	–	–	–
TN	0.049	0.44	0.049	0.073	0.403	0.092	0.073	0.138	0.433	–	–
TT	0.049	0.299	0.049	0.049	0.347	0.049	0.049	0.049	0.780	0.330	–
V	0.092	0.69	0.049	0.049	0.299	0.049	0.092	0.049	0.073	0.454	0.299

The values represent the p adjusted values after Bonferroni correction, obtained using random permutations.

**Figure 3:**
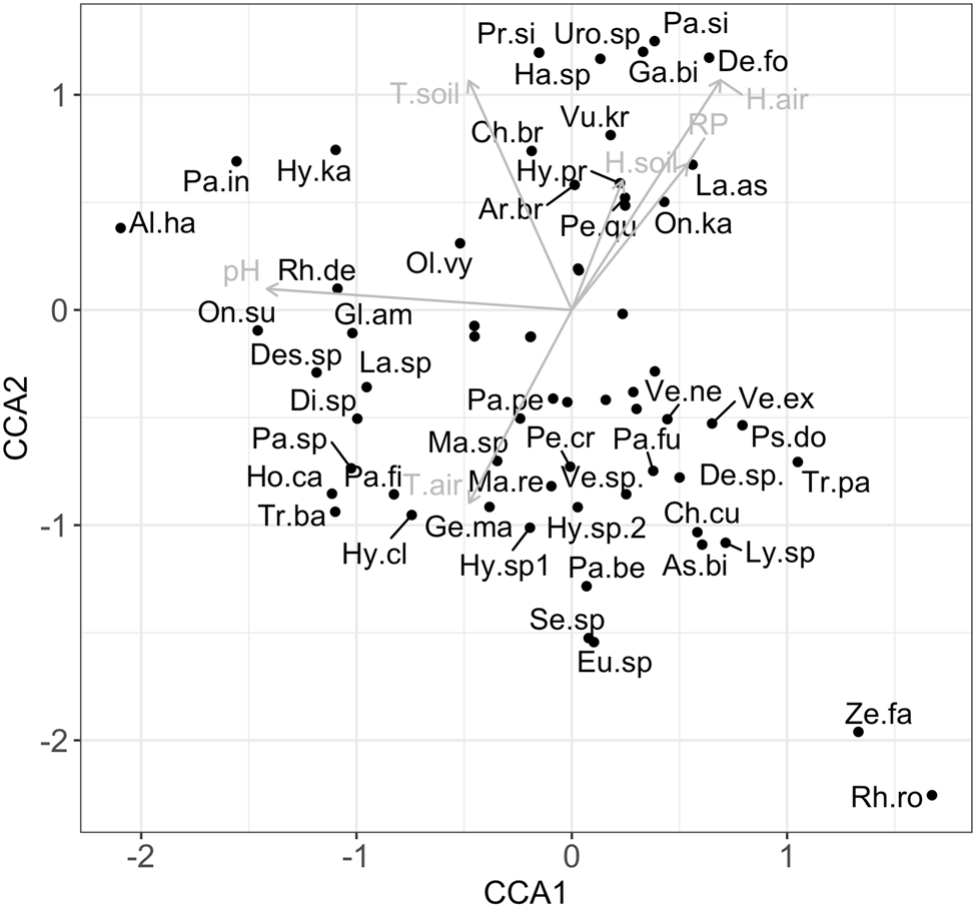
Biplots of the canonical correspondence analysis model of species abundance matrix in relation environmental variables. Length and direction of arrows indicate the relative importance and direction of change in the environmental variables. Variables are: T-soil and T-air, H-soil and H-air, pH, RP. Species names were abbreviated using the initials of the genus and species name (abbreviations are reported in Tables 3 and 4).

The CCA model including all the environmental variables explained a total of 52.00% of the overall variation in species composition, of which 26.01% was explained by the first axis and 25.9% by the second axis, respectively. The first canonical axis was highly correlated with pH (− 0.944) whereas the second canonical axis was correlated with T-soil (0.710), T-air (− 0.598) and H-air (0.712). The biplot classification on the two first axes showed species from the upper right quadrant were associated with H-soil, Rp, T-soil and H-air. The lower quadrant contains species associated with T-air. Species associated with pH were placed in the upper left quadrant (Fig. 3).

The ANOVA permutation test for CCA showed that all the environmental variables were significantly related to community assemblage (Supplementary Appendix 5).

**Figure 4:**
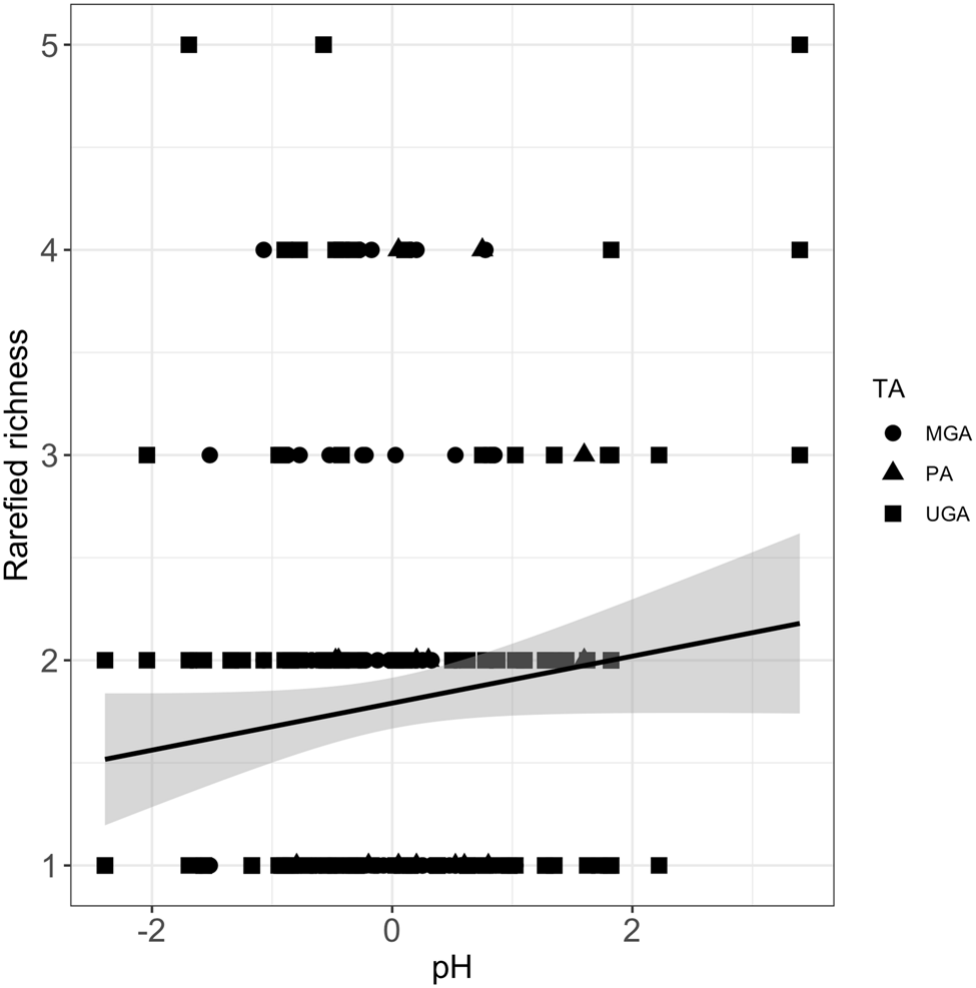
Linear regression between species richness, grouped by management scenarios (MS): protected areas (PA), managed green areas and (MGA) and unmanaged green areas (UGA), and pH standardised to have mean zero and unit variance. The shaded area represents the confidence interval.

Based on species richness, the model selection using AICc indicated one highly supported model that included the pH (Table 6). The second best supported model included the MS. Species richness significantly increased with pH (*F*_[1,231]_ = 4.00, *p* = 0.04) for all management scenarios (Fig. 4). No significant differences in species richness were found among the UGA (*F*_[2,106]_ = 0.9, *p* = 0.533).

**Table 6:** Akaike statistics for model including the species richness, the management scenario and the environmental variables, AIC (Akaike’s Information Criterion) differences (ΔAICc) and Akaike weights (wi) were used to rank models relative to the best model (minimum AIC).

Model	Covariates	K	AIC_c_	ΔAIC_c_	w_i_	LL
Mod6	pH	4	696	0	0.82	− 344
Mod1	MS	5	700	4.17	0.1	− 345
Mod10	MS + pH	6	701	5.96	0.04	− 345
Mod3	T.air + RP	5	704	8.82	0.01	− 347
Mod4	H.soil + RP	5	704	8.85	0.01	− 347
Mod2	Tsoil + RP	5	704	8.86	0.01	− 347
Mod5	H.air + RP	5	705	9.04	0.01	− 347
Mod8	MS + T.air + RP + H.air + pH	9	714	18.87	0.00	− 348
Mod9	MS + T.soil + RP + H.soil + pH	9	717	21.04	0.00	− 349
Mod7	MS + T.soil + RP + H.soil + pH + T.air + H.air	11	723	27.93	0.00	− 351

*K* number of parameters, *LL* log likelihood.

Focussing on individual Bucharest parks, the highest number of identified species and number of individuals was recorded in TN park (17 species with 157 individuals), in comparison with V, CL or FL, PL areas, where the values were lowest (number of species between 8 and 10 and number of individuals between 25 and 50 individuals). These results are confirmed by the Shannon index of diversity, which had the highest value in TN (H = 2.085) and lowest in V or TT urban parks (H = 1.696 or H = 1.572). The maturity index of predatory mite communities had its highest values in C and V (0.66 and 0.61). In contrast, communities from CL, TN and FL had a maturity index of only 11 or 10 (Table 3).

Turning to the dominance index, the mite communities from TT, V and PL are represented by a few dominant species: *Pergamasus quisquiliarum* and *Arctoseius cetratus* in TT, *Rhodacarellus silesiacus* in V, and *Hypoaspis aculeifer* in all three ecosystems. In the remaining green areas studied, representation of remaining species was almost equitable. This fact was indicated by the equitability index, characteristic for each urban area (Table 3).

Comparing the taxonomic structure of the mite communities, we observed that each managed urban green area was defined by some characteristic species i.e. (a) for the natural park: *Pergamasus crassipes* and *Parasitus fimetorum*, (b) for metropolitan parks: *Cherosieus bryophilus* and *Leioseius minusculus*, (c) for municipal parks: *Paragamasus similis*, *Protogamasellus singularis* and *Olopachys vysotskajae*, and (d) for district parks: *Pergamasus laetus*, *Parasitus beta, Pseudolaelaps doderoi* and *Laelaps astronomica*.

In the different types of managed parks in Bucharest, we observed that in MtP, the number of species as well as numerical densities is higher than in DtP. In MtP the average value of soil moisture is higher than in DtP (15.63 in comparison with 10.84). The mean value of soil temperature is higher in MtP (18.06 °C) that in DtP (13.41 °C). These environmental variables could influence the mite communities from the two types of managed urban areas.

Making an ecological analysis of the mite communities from UGA areas, the highest number of identified species was recorded in B park (22 species), as well as the Shannon index of diversity (H = 2.791) and equitability (J = 0.903). The highest value for numerical abundance was obtained in park F (110 individuals). In contrast, park P has lower values for all these parameters, with the exception of dominance, whose value was D = 0.294 and of maturity index (M = 0.71) (Table 4).

In UGA, the most common species were *Veigaia planicola, Hypoaspis aculeifer, Rhodacarellus silesiacus* (present in all parks), *Pergamasus crassipes, Parasitus fimetorum, Veigaia exigua, Veigaia nemorensis, Macrocheles recki, Onchodellus karawaiewi* and *Alliphis siculus* (identified in three of the parks investigated) (Table 4).

Indicator species analysis identified two characteristic mite species to UGA, i.e., *Veigaia planicola* (Ind.Val = 25.8%, p = 0.006) and *Macrocheles recki* (Ind.Val = 22.2%, p = 0.015) and one species to MGA and PA combination, i.e., *Pergamasus quisquiliarum* (Ind.Val = 30.1%, p = 0.002).

## Discussion

Comparing the present research with data obtained from other studies in Europe on the Mesostigmata of urban soils, the number of species identified in Bucharest is comparable with totals obtained in Latvia and Poland, in such different habitats as roadsides, greenery, parks and urban forests (25–51 species), but is higher than that for mite communities in a botanical garden in Hungary (12 species)^40,42,45,47^. However, the number of recorded species is lower than suburban and urban green areas from Poland (62–68 species), urban grasslands from Latvia or cemeteries and botanical garden in Slovakia (123 species, in three years of study)^34,35,38,39,43^.

Analysing the above presented results, we observed that management intensity in urban green areas, from Bucharest-Romania, impacts mesostigmatid mite diversity and community composition. The effect of management intensity on mesostigmatid mite diversity was highlighted by the results of pairwise comparison of species communities that revealed significant differences between MGA and UGA areas. The number of recorded species in UGA is higher than that for MGA. The Romanian results agree with those obtained in Latvia for Riga city, where there was a high diversity of soil invertebrates in the disturbed urban forest habitats, but undisturbed soils harbour a greater species richness of mites than disturbed soils^40^.

The management type of the urban green areas influenced the species composition as well. Species composition differs in the three management scenarios for urban green areas. About 27.54% of the total number of species were characteristic of MGA, with *Pergamasus quisquiliarum, Dendrolealaps foveolatus* and *Gamasellus bicolor* having the highest recorded abundance. Other research has indicated that *Pergamasus quisquiliarum* is a species with affinity for parks and street green areas in Warsaw, Poland. *Dendrolealaps foveolatus* and *Gamasellus bicolor* have been identified in urban forest in Riga city and in suburban green areas in Warsaw^35,45^.

In UGA, 47.83% are species identified only in these parks. In these mite communities, species from the Macrochelidae and Pachylaelapidae families were best represented, with *Macrocheles recki, Aliphis halleri* and *Parasitus fimetorum* most numerous. These species were also associated with industrial and post-industrial areas in Poland^38^.

Comparing the mite communities of PA with those from the remaining MGA, we observed not only the lowest number of species and numerical densities in PA but also the lowest value of the Shannon index of diversity (H = 1.696). The same situation is found if we compare PA mite communities with all communities from unmanaged urban green areas. These results are supported by the MANOVA pairwise comparisons of species communities among sites (Table 5). Analysing abiotic factors, we observed that in the PA, the highest mean values of soil and air temperature were recorded (16.87 °C and 30.02 °C), potentially affecting the structure of mite communities. Despite the fact that this PA (Văcăreşti) is wetland, higher temperatures during summer and autumn cause high evapotranspiration and water depletion at depth in the soil^72^.

Comparison between species communities from MGA revealed that those from DrP and MtP were significantly different (according to PERMANOVA test). In managed types of district park green areas the number of species as well as numerical densities are higher than in metropolitan parks. Some environmental variables are significantly different between parks e.g. soil moisture and temperature. There are also differences in community composition structure. All these differences are correlated with specific environmental conditions. Higher soil humidity and temperature provided more favourable environmental conditions for predatory soil mites in metropolitan parks. Drought or lower soil humidity has a negative impact on soil microarthropod fauna, both in general and on mites specifically^73^.

Using the presented information, based on the obtained results from this research, we could propose that the future new created urban parks should be designed taking into account the natural/unmanaged model. This means that, from acarological point of view, the autochthone vegetation and native soils constitute a proper habitat for Mesostigmata species. In unmanaged urban green areas, the ecological process are natural and undisturbed (not anthropized as in managed parks) and these ecosystems will follow the natural ecological succession. If we put into discussion the relation between ecological characteristics of the managed urban green areas and Mesostigmata fauna, we consider that their management could be reconsidered. This reconsideration means to use native species of plant, trees and soil, reach in organic matter (not indigenous brought from Asia or Central America, usually accompanied by the alochtonous soil and other invasive species).

Research studies from all over the world revealed that the Mesostigmata communities correlated with environmental variables could constitute a very useful biological tool to characterized the ecological quality of terrestrial ecosystems and the quality of soils^9,10,18,21,25–27,29–31,33^. In the case of urban green areas from Bucharest, under different management scenarios, their ecological characterization was made taking into account the variation of the environmental parameters, in correlation with spatial dynamics of the communities’ parameters of Mesostigmata fauna (species diversity and numerical abundance).

Analysing the environmental parameters, the highest average value of soil moisture (13.16%) was recorded in MGA areas where the soil penetration resistance is higher (RP = 1.8). However, the differences between mean values of soil moisture for all the green areas are not significant (p = 0.334), due to the fact the soils throughout Bucharest are mainly clay (Table 1). Because of their very small particle size and consequent large surface area, clays are able to retain greater amounts of water than sandy or most anthropogenic soils^74^. Other research studies revealed that there was a strong correlation between soil penetration resistance, pore space, soil moisture and quantity of organic matter^75,76^.

In general, an increased value of RP means a decreased quantity of organic matter, a lower value of soil moisture and reduced pore space. Although soil moisture in our study is higher in MGA than in UGA or PA, it is possible that the lack of organic matter is the main limiting factor for species diversity. However, clay particles from MGA soils had a much greater tendency to stick together than sands (which were present mainly in UGA), and not only did soil moisture have recorded high values but also the RP is higher^74^.

In the investigated plots, especially those in UGA, the dominant species of Mesostigmata are predatory mites. Their presence is linked to organic matter (correlated with pH), which represents a trophic reservoir and favourable habitat for other invertebrate groups—a relationship supported by the higher values of the soil maturity index^15,28,77,78^. The relationship between clay soil and pH is well established. Clays have a large specific surface and are predominantly negatively charged, retaining nutrients against leaching and buffering the soil against extreme pH changes^79^. It has also been discovered that on basic soil some species of bacteria develop, with these two variables being linearly correlated^80^. Bacterial community development constitutes a favourable factor for the soil invertebrates that represent the food source for predatory mites^9,77^. This may also explain the linear relation between pH value and species richness obtained in our research.

Soil structure is undisturbed in UGA compared with MGA; in MGA there is some modification every year due to the establishing of different ornamental plants. According to specialists^81^, some soils in Bucharest were identified that, although less modified, nonetheless received (and were still receiving) mainly negative impacts caused by daily household or industrial activities. Soils of this type were found in those green spaces less modified by urbanism, and soils from peripheral and suburban areas.

If we consider the influence of the environmental variables on all the mite species identified, we observed that species such as *Dendrolealaps foveolatus*, *Pergamasus quisquiliarum, Laelaps astronomica, Hypoaspis praesternalis* and *Onchodellus karawaiewi* were influenced by soil moisture and air humidity, soil temperature and RP. In Europe, these species have been identified mostly in grasslands, grassy arable fallows or urban habitats, their communities being correlated with soil moisture (*Onchodellus karawaiewi* being a euryhygrophilous species) or organic matter content. In our study, this explains the relationship between such mites and RP^20,25,44,60,79^.

Three species (*Rhodacarus denticulatus, Olopachys suecicus* and *Glyptholaspis americana*) were influenced by soil pH. Soil pH itself is influenced by different vegetation types, including those from urban green areas^20,82^. *Olopachys suecicus* is eurytopic, with wide ecological plasticity, able to colonise poorly shaded habitats (urban parks, scrub, grasslands). *Glyptholaspis americana* is cosmopolitan and mostly found in acid habitats such as dung or leaf litter^58^.

Rather surprisingly, in these urban ecosystems air temperature influenced the composition of mite communities indirectly. In such ecosystems, the most striking difference from surface habitats was that the moisture/humidity and temperature extremes were much less pronounced in the soil. Similar patterns have been identified in other habitat types, such as screes^83^.

Spatial dynamics of the investigated parameters of soil mite communities related to environmental factors could constitute a response of this invertebrate group to the specific conditions of urban green areas under different types of management scenario.

## Conclusions

The presented study established the effect of management type and of environmental variables on the structure, abundance and species richness of soil mites (Acari: Mesostigmata). For the first time in Romania, an intensive study on urban Mesostigmata fauna has been made. Twelve urban areas were characterised by specific environment conditions and mite communities. Six environmental factors were investigated (soil and air temperature, soil moisture and atmospheric humidity, soil pH, soil penetration resistance). In total 480 soil samples were analysed for the soil fauna, as well for the abiotic factors.

Examining the mean values of environmental variables, we observed that soil temperature, air temperature, air humidity and soil penetration resistance showed significant differences between all three types of managed urban green area. All environmental variables, especially soil pH, significantly influenced the mite communities. The MGA areas were characterised by the highest mean values of air and soil moisture, the PA had the highest mean values of air and soil temperature, pH and the UGA by the lowest values of these parameters. From all collected soil samples, 68 Mesostigmata species were identified, with 790 individuals and 49 immatures. In terms of the number of species and numerical abundance in relation to management regimes and to the specific investigated environmental conditions, differences in mesostigmatid mite communities were recorded between managed green areas (MGA) and unmanaged green areas (UMG). In comparison with MGA, UGA were characterised by higher values of the community parameters (Shannon diversity, dominance and equitability), as well as by the highest values of the soil maturity index. Comparative analysis between the three types of MGA revealed that the species communities from metropolitan parks were richer than those from district parks.

The investigated indices that showed different values in relation to environmental variables demonstrate important links between mite communities in specifically urban ecosystems that are under anthropogenic pressure. On the other hand, we consider that unmanaged urban green areas as “hotspots” of Mesostigmata diversity. This will constitute an argument of the usage of native vegetation and soil, for creation of new urban parks or for management of those existing ones.

## Supplementary Information

Supplementary Information 1.

Supplementary Information 2.

Supplementary Information 3.

Supplementary Information 4.

Supplementary Information 5.

## Data Availability

The data sets analysed during the current study are available from the corresponding author on reasonable request.

## References

[1] Bremner J, Frost A, Haub C, Mather M, Ringheim K (2010). World population highlights: Key findings from PRB’s 2010 world population data sheet. Popul. Bull..

[2] McGranahan, G., Marcotullio, P. Urban systems. In *Ecosystems and Human Well-being: Current State and Trends. Volume I* (eds. Hassan, R., Scholes, R., Ash, N.) 795–825 (Island Press, Washington, DC, 2005).

[3] Robrecht, H. & Lorena, L. Ecosystem services in cities and public management. In *The Economics of Ecosystems and Biodiversity for Local and Regional Policy* (ed Wittmer, H.) 60–80 (Progress Press, 2010).

[4] Adhikari K, Hartemink AE (2016). Linking soils to ecosystem services—A global review. Geoderma.

[5] Millennium Ecosystem Assessment Panel. Ecosystems and Human Well-being: Synthesis. Washington, DC: Island Press. https://www.millenniumassessment.org/en/index.html (2005).

[6] McDonald, R.I., Marcotullio, P.J. & Güneralp, B. Urbanization and Global Trends in Biodiversity and Ecosystem Services. In *Urbanization, Biodiversity and Ecosystem Services: Challenges and Opportunities. A Global Assessment *(eds. Elmqvist, T. *et al.*) 31–52 (Springer, Dordrecht, 2013).

[7] Anthrop M (2000). Changing patterns in the urbanized countryside of Western Europe. Landsc. Ecol..

[8] Coleman, D.C. & Wall, D.H. Soil fauna: Occurrence, biodiversity, and roles in ecosystem function. In *Soil Microbiology, Ecology and Biochemistry* (ed Paul, E.) 111–149 (Academic Press, Waltham, 2015).

[9] Dirilgen T, Arroyo J, Dimmers WJ, Faber J, Stone D (2016). Mite community composition across a European transect and its relationships to variation in other components of soil biodiversity. Appl. Soil Ecol..

[10] Culliney TW (2013). Role of arthropods in maintaining soil fertility. Agriculture..

[11] Krantz, G. W. & Walter, D. E. *A manual of Acarology*. (ed. Texas Tech University Press, USA) 98–100 (Krantz & Walter, 2009).

[12] McIntyre NE (2000). Ecology of urban arthropods: A review and a call to action. Ann. Entomol. Soc. Am..

[13] Jones EL, Leather SR (2012). Invertebrates in urban areas: A review. Eur. J. Entomol..

[14] Koehler HH (1999). Predatory mites (Gamasina, Mesostigmata). Agric. Ecosyst. Environ..

[15] Gulvik ME (2007). Mites (Acari) as indicators of soil biodiversity and land use monitoring: A review. Pol. J. Ecol..

[16] Salmane I, Brumelis G (2010). Species list and habitat preference of Mesostigmata mites (Acari, Parasitiformes) in Latvia. Acarologia..

[17] Kaczmarek S, Marquardt T, Falenczyk-Kozirog K (2011). Diversity of the Mesostigmata (Acari) in tree-hollows of selected deciduous tree species. Biol. Lett..

[18] Madej G, Barczyk G, Gawenda J (2011). Importance of microhabitats for preservation of species diversity, on the basis of mesostigmatid mites (Mesostigmata, Arachnida, Acari). Pol. J. Environ. Stud..

[19] Huhta V, Pietikäinen AS, Penttinen R (2012). Importance of dead wood for soil mite (Acarina) communities in boreal old-growth forests. Soil Organ..

[20] Wissuwa J, Salamon JA, Frank T (2012). Effects of habitat age and plant species on predatory mites (Acari, Mesostigmata) in grassy arable fallows in Eastern Austria. Soil Biol. Biochem..

[21] Manu, M. Structure and dynamics of the predatory mites (Acari: Mesostigmata- Gamasina) from the central parks and forest ecosystems from/near Bucharest. In *Species Monitoring in the Central Parks of Bucharest* (ed. Onete, M.) 68–78 (Ars Docendi, Universitatea Bucureşti, 2008).

[22] Manu, M., Szekely, L., Vasiliu, Oromulu, L., Bărbuceanu, D., Honciuc, V. *et al.* Bucharest. In *Vertebrates and Invertebrates of European Cities: Selected Non-Avian Fauna* (ed. Kelcey, J.G.) 257–322 (Springer Science+Business Media LLC, New York, 2015).

[23] Manu M, Băncilă RI, Onete M (2018). Importance of moss habitats for mesostigmatid mites (Acari: Mesostigmata) in Romania. Turk. J. Zool..

[24] Klarner B, Maraun M, Scheu S (2013). Trophic diversity and niche partitioning in a species rich predator guild—natural variations in stable isotope ratios (13C/12C, 15N/14N) of mesostigmatid mites (Acari, Mesostigmata) from Central European beech forests. Soil Biol. Biochem..

[25] da Groot AG, Jagers op Akkerhuis GJAM, Dimmers WJ, Charrier X, Faber JH (2016). Biomass and diversity of soil mite functional groups respond to extensification of land management, potentially affecting soil ecosystem services. Front. Environ. Sci..

[26] Manu M, Iordache V, Băncilă RI, Bodescu F, Onete M (2016). The influence of environmental variables on soil mite communities (Acari: Mesostigmata) from overgrazed grassland ecosystems—Romania. Ital. J. Zool..

[27] Meehan ML, Zhuoyan Song Z, Proctor H (2018). Roles of environmental and spatial factors in structuring assemblages of forest-floor Mesostigmata in the boreal region of Northern Alberta, Canada. Int. J. Acarol..

[28] Kamczyc J, Skorupski M, Dyderski MK, Gazda A, Hachułka M (2018). Response of soil mites (Acari, Mesostigmata) to long-term Norway spruce plantation along a mountain stream. Exp. Appl. Acarol..

[29] Santorufo L, Van Gestel CM, Rocco A, Maisto G (2015). Soil invertebrates as bioindicators of urban soil quality. Environ. Pollut..

[30] N’Dri JK, Hance T, Andr,é HM, Lagerlöf J, Tondoh JE (2016). Microarthropod use as bioindicators of the environmental state: Case of soil mites (Acari) from Côte d’Ivoire. J. Anim. Plant Sci..

[31] George PBL, Keith AM, Creer S, Barrett GL, Lebron I (2017). Evaluation of mesofauna communities as soil quality indicators in a national-level monitoring programme. Soil Biol. Biochem..

[32] Manu M, Onete M, Băncilă RI (2018). The effect of heavy metals on mite communities (Acari: Gamasina) from urban parks—Bucharest, Romania. Environ. Eng. Manag. J..

[33] Spiller MS, Spiller C, Garle J (2018). Arthropod bioindicators of environmental quality. Revista Agroambiente..

[34] Niedbała W, Błaszak C, Błoszyk J, Kaliszewski M, Kazmierski A (1982). Soils mites (Acari) of Warsaw and Mazovia. Memorabilia Zool..

[35] Niedbała W, Błoszyk J, Kaliszewski M, Kazmierski A, Olszanowski Z (1990). Structure of soil mite (Acari) communities in urban green of Warsaw. Fragmenta Faunistica..

[36] Pouyat RV, Parmelee RW, Carreiro MM (1994). Environmental effects of forest soil-invertebrate and fungal densities in oak stands along an urban-rural land use gradient. Pedobiologia.

[37] Minor MA, Cianciolo JM (2007). Diversity of soil mites (Acari: Oribatida, Mesostigmata) along a gradient of use types in New York. Appl. Soil Ecol..

[38] Skorupski M, Horodecki P, Jagodziński AM (2013). Roztocze z rzędu Mesostigmata (Arachnida, Acari) na terenach przemysłowych i poprzemysłowych w Polsce. (Mite species of Mesostigmata order (Arachnida, Acari) in industrial and post-industrial areas of Poland). Nauka Przyr. Technol..

[39] Minova S, Jankevica L, Salmane I, Èekstere G (2015). Preliminary studies on microbial biomass and the microarthropod community as soil health and quality indicators in urban grasslands, Rîga as an example. Proc. Latvian Acad. Sci. Sect. B..

[40] Telnov, D. & Salmane, I. Ecology and diversity of urban pine forest soil invertebrates in Rîga, Latvia. *Proc. Latvian Acad. Sci. Sect. B Nat.* **69**(3), 120–131 (2015).

[41] Napierała, A., Skwierczyñski, F. & Jankowiak, A. Materials to knowledge of Uropodina (Acari: Mesostigmata) of Poznań District. *Badania Fizjograficzne R. I Seria C Zoologia.* **C51**, 7–19 (2010).

[42] Kontschán J, Ács A, Wang GQ, Neményi A (2015). New data to the mite fauna of Hungarian bamboo plantations. Acta Phytopathol. Entomol. Hung..

[43] Fend’a, P. & Hruzova, K. Mites (Acari, Mesostigmata) in urban green of Bratislava (Slovakia) In *8th Symposium of the European Association of Acarologist* (ed Universitat Politecnica de Valencia) 41 (Book of Abstract, 2016).

[44] Hrúzová, K. & Fend’a, P. First record of Parasitus americanus (Berlese, 1905) and Cornigamasus ocliferius Skorupski and Witaliński, 1997 (Acari: Mesostigmata: Parasitidae) from Slovakia. *Check List*. **13**(4), 239–243 (2017).

[45] Salmane I (2018). Soil microarthropods (Acari, Collembola) in the Rīga city habitats. Environ. Exp. Biol..

[46] Błoszyk J, Klimczak I, Leśniewska M (2006). Phoretic relationships between Uropodina (Acari: Mesostigmata) and centipedes (Chilopoda) as an example of evolutionary adaptation of mites to temporary microhabitats. Eur. J. Entomol..

[47] Napierała A, Książkiewicz Z, Leśniewska M, Gwiazdowicz DJ, Mądra A, Błoszyk J (2015). Phoretic relationships between uropodid mites (Acari: Mesostigmata) and centipedes (Chilopoda) in urban agglomeration areas. Int. J. Acarol..

[48] Mizser S, Nagy L, Tóthmérész B (2016). Mite infection of *Carabus violaceus* in rural forest patches and urban parks. Period. Biol..

[49] Honciuc V, Manu M (2010). Ecological study on the edaphically mite’s populations (Acari: Mesostigmata—Gamasina: Oribatida) in urban areas from Romania. Rom. J. Biol. Zool..

[50] Manu M, Honciuc V (2010). Rang correlations at the level of the predator and the decomposer populations soil mites (Acari: Mesostigmata-Gamasina, Oribatida) from central parks of Bucharest city, Romania. Acta Entomol. Serb..

[51] Manu M, Honciuc V (2010). Ecological research on the soil mite’s populations (Acari: Mesostigmata-Gamasina, Oribatida) from forest ecosystems near Bucharest city. Rom. J. Biol. Zool..

[52] Iojă CI, Rozylowicz L, Pătroescu M, Niţă MR, Vânau GO (2011). Dog walkers’ vs other park visitors’ perceptions: The importance of planning sustainable urban parks in Bucharest, Romania. Landsc. Urban. Plan..

[53] Pătroescu M, Ioja C, Necsuliu R, Brailescu C (2004). The quality of oxygenating surfaces. The green areas of Bucharest. A case studies. Rev. Roum. Geogr..

[54] Trzyna, T. *Urban Protected Areas: Profiles and best practice guidelines*. Best Practice Protected Area Guidelines Series No. 22, Gland, Switzerland: IUCN (2014).

[55] Ghiliarov, M.S. & Bregetova, N.G. Opredeliteli obitayushchikh v pochve kleshcheĭ Mesostigmata. (Akademia Nauk USSR, Zoologicheskiĭ Institut Evolyucionoĭ Morfologii i Ekologii zhivotnikh im A.H. Savertova, Izd. Nauka, Leningrad, 1977).

[56] Hyatt KH (1980). Mites of the subfamily Parasitinae (Mesostigmata: Parasitidae) in the British Isles. Bull. Br. Mus. Nat. Hist. Zool..

[57] Karg, W. Acari (Acarina), Milben Parasitiformes (Anactinochaeta) Cohors Gamasina Leach. **59**, 1–513 (1993).

[58] Mašán, P. *Macrochelid Mites of Slovakia (Acari, Mesostigmata, Macrochelidae)* (Institute of Zoology, Slovak Academy of Science, Bratislava, 2003).

[59] Mašán P (2003). Identification key to Central European species of Trachytes (Acari: Uropodina) with redescription, ecology and distribution of Slovak species. Eur. J. Entomol..

[60] Mašán P, Fenďa P (2004). Zerconid Mites of Slovakia (Acari, Mesostigmata, Zerconidae.

[61] Mašán P (2007). A Review of the Family Pachylaelapidae in Slovakia with Systematics and Ecology of European Species (Acari: Mesostigmata: Eviphidoidea).

[62] Mašán P, Fenďa P, Mihál I (2008). New edaphic mites of the genus Veigaia from Slovakia and Bulgaria, with a key to the European species (Acari, Mesostigmata, Veigaiidae). Zootaxa..

[63] Mášan P, Halliday B (2010). Review of the European genera of Eviphididae (Acari: Mesostigmata) and the species occurring in Slovakia. Zootaxa..

[64] Oksanen, J., Blanchet, G.F., Friendly, M., Kindt, R., Legendre, P., McGlinn, D., Minchi, P.R., O'Hara, R.B., Simpson, G.L., Solymos, P., Stevens, M.H., Szoecs, E., Wagner, H. *Vegan: Community Ecology Package. R package version 2.4–0.* https://cran.r-project.org/package=RVAideMemoire (2019).

[65] Herve, ´ M. *RVAideMemoire: Testing and Plotting Procedures for Biostatistics*. R package version 0.9-66. https://CRAN.R-project.org/package=RVAideMemoire (2017).

[66] Burnham KP, Anderson DR (2002). Model Selection and Multimodel Inference: A Practical Information—Theoretic Approach.

[67] Johnson JB, Omland KS (2004). Model selection in ecology and evolution. Trends Ecol. Evol..

[68] Hammer Ø, Harper DAT, Ryan PD (2001). PAST: Paleontological statistics software package for education and data analysis. Palaeontol. Electron..

[69] Ruf A (1998). A maturity index for predatory soil mites (Mesostigmata, Gamasina) as an indicator of environmental impacts of pollution of forest soils. Appl. Soil Ecol..

[70] De Caceres, M., Legendre, P. *Associations Between Species and Groups of Sites: Indices an Statistical Inference*. Ecology. http://sites.google.com/site/miqueldecaceres (2009). 10.1890/08-1823.1 20120823

[71] Dufrêne M, Legendre P (1997). Species assemblages and indicator species: The need for a flexible asymmetrical approach. Ecol. Monog..

[72] Zaharia V, Găitănaru D (2018). Aspects of water budget in Văcăreşti wetland. Math. Model. Civ. Eng..

[73] Xu G-L, Kuster TM, Günthardt-Goerg MS, Dobbertin M, Li M-H (2012). Seasonal exposure to drought and air warming affects soil collembola and mites. PLoS ONE.

[74] Gülser C, Candemir F (2012). Changes in penetration resistance of a clay field with organic waste applications. Eurasian J. Soil Sci..

[75] Bergamin AC, Vitorino ACT, Souza FR, Venturoso LR, Luara PP (2015). Relationship of soil physical quality parameters and maize yield in a Brazilian Oxisol. Chil. J. Agric. Res..

[76] Jones MF, Arp PA (2017). Relating cone penetration and rutting resistance to variations in forest soil properties and daily moisture fluctuations. Open J. Soil Sci..

[77] Ekschmitta K, Liub M, Vettera S, Foxa O, Wolters V (2005). Strategies used by soil biota to overcome soil organic matter stability—why is dead organic matter left over in the soil?. Geoderma.

[78] Gulvik ME, Błoszyk J, Austad I, Bajaczyk R, Piwczyński D (2008). Abundance and diversity of soil microarthropod communities related to different land use regime in a traditional farm in Western Norway. Pol. J. Ecol..

[79] Newman ACD (1984). The significance of clays in agriculture and soils. Philos. Trans. R. Soc. Lond A..

[80] Shen C, Xiong J, Zhang H, Youzhi Y, Lin X, Li X, Liang W, Chu H (2013). Soil pH drives the spatial distribution of bacterial communities along elevation on Changbai Mountain. Soil Biol. Biochem..

[81] Lăcătuşu R, Lăcătuşu AR, Lungu M, Breaban IG (2008). Macro- and microelements abundance in some urban soils from Romania. Carpath. J. Earth Environ. Sci..

[82] Chikoski JM, Ferguson SH, Meyer L (2006). Effects of water addition on soil arthropods and soil characteristics in a precipitation-limited environment. Acta Oecol..

[83] Nitzu E, Nae A, Băncilă RI, Popa I, Giurgincă A, Plăiasu R (2014). Scree habitats: Ecological function, species conservation and spatial-temporal variation in the arthropod community. Syst. Biodivers..

