# Supplementary material for: Soil mite communities (Acari: Mesostigmata) as indicators of urban ecosystems in Bucharest, Romania
Source: Sci Rep. 2021 Feb 15;11:3794. doi: 10.1038/s41598-021-83417-4 (PMC7884781; doi:10.1038/s41598-021-83417-4)
Supplement: Supplementary file 1 — Supplementary Information 1. [file 41598_2021_83417_MOESM1_ESM.docx]

Soil mite communities (Acari: Mesostigmata) as indicators of urban ecosystems in Bucharest, Romania

Manu M.^1*#^, Băncilă R.I.^2,3#^, Bîrsan C.C.^1^, Mountford O.^4^, Onete M.^1^

^1^Romanian Academy, Institute of Biology Bucharest, Department of Ecology, Taxonomy and Nature Conservation, street Splaiul Independenţei, no. 296, zip code 0603100, PO-BOX 56-53, fax 040212219071, tel. 040212219202, Bucharest, Romania, email: minodoramanu@gmail.com; ciprian.birsan@ibiol.ro, marilena.onete@gmail.com

^2^Faculty of Natural Sciences, University Ovidius Constanţa, Constanţa, Romania

^3^Department of Biospeleology and Soil Edaphobiology, “Emil Racoviţă” Institute of Speleology, Romanian Academy, 13 Septembrie Road, No. 13, 050711, Bucharest, Romania, email: bancila_ralucaioana@yahoo.com

^4^Centre for Ecology and Hydrology, Maclean Building, Benson Lane, Crowmarsh Gifford, Wallingford, Oxfordshire, OX10 8BB, UK, email: om@ceh.ac.uk

Corresponding author email: minodoramanu@gmail.com

^#^ The authors had an equal contribution to the production of this article.

Appendix 1

Detailed description of the investigated urban areas in Bucharest (2017)

| **Urban protected area** | **Văcăreşti**  N: 44°23'53.5"  E: 26°08'18.4"  65 m  183 ha  Protected area  City centre  Tree layer*: Salix alba, Typha angustifolia, Morus alba.*  Shrub layer*: Sambucus ebulus*  Herbaceous layer*: Phragmites australis, Alisma plantago-aquatica, Poa cf trivialis, P. palustris, Juncus articulatus, J. bufonius, Epilobium hirsutum, Plantago lanceolata , Trifolium pratense, Juncus subnodulosus, Dactylis glomerata, Daucus carota, Bromus cf sterilis, Vicia cracca, Vicia cf sativa, Galium sp., Dipsacus fullonum.*  Clay | | |
| --- | --- | --- | --- |
| Geographical coordinates: |  |  |  |
| Altitude |  |  |  |
| Area |  |  |  |
| Park category |  |  |  |
| Location within city |  |  |  |
| Vegetation description (dominant plant species) |  |  |  |
| Type of soil |  |  |  |
| **Managed urban areas** | **Tineretului** | **Titan** | **Plumbuita** |
| Geographical coordinates: | N: 44°24'31.0"  E: 26°06'38.1" | N: 44°25'33.7"  E: 26°09'22.2" | N: 44°28'05.3"  E: 26°08'16.9" |
| Altitude | 62 m | 71 m | 68 m |
| Slope | 5^0^ | 5^0^ | 0 |
| Exposure | South-East | South-East | - |
| Area | 94 ha | 48 ha | 67 ha |
| Park category | Metropolitan | Metropolitan | Municipal |
| Location within city | City centre | City centre | City centre |
| Vegetation description (dominant plant species) | Tree layer*: Populus cf x canadensis, Acer negundo, Casuarina sp., Metasequoia glyptostroboides.*  Herbaceous layer*: Dactylis glomerata, Arctium sp., Lolium perenne, Poa spp., Cirsium arvense.* | Tree layer*: Platanus x hispanica*, *Aesculus hippocastanum, Populus nigra “Italica”, Salix alba, Salix babylonica, Typha sp.*  Herbaceous layer*: Phragmites australis, Lolium perenne, Poa spp., Bellis perennis, Trifolium repens.* | Tree layer*: Salix babylonica “Pekingensis”, Platanus x hispanica*, *Populus nigra “Italica”, Acer negundo.*  Shrub layer: *Elaeagnus angustifolia.*  Herbaceous layer: *Lolium perenne,, Poa spp., Dactylis glomerata, Alopecurus pratensis, Plantago lanceolata, Trifolium repens, Trifolium pratense, Arctium sp., Elytrigia repens, Cichorium intybus.* |
| Type soil | Clay | Clay | Clay |
|  | **Carol** | **Floreasca** | **Crângaşi** |
| Geographical coordinates: | N: 44°24'47.6"  E: 26°05'44.0" | N: 44°28'00.3"  E: 26°05'58.4" | N: 44°27'08.9"  E: 26°02'23.0" |
| Altitude | 66 m | 77 m | 79 m |
| Slope | 10^0^ | 0 | 20^0^ |
| Exposure | South-East | - | North-East |
| Area | 29.3 ha | 7.8 ha | 8 ha |
| Park category | Municipal | District | District |
| Location within city | City centre | City centre | City centre |
| Vegetation description (dominant plant species) | Tree layer*: Aesculus cf hippocastanum, Populus nigra “Italica”, Platanus x hispanica, Alnus glutinosa, Salix babylonica*, *Sambucus nigra, Fraxinus excelsior, Tilia x europaea, Robinia pseudoacacia, Aesculus hippocastanum, Salix alba, Salix fragilis, Prunus cf avium, Acer cf pseudoplatanus, Metasequoia glyptostroboides, Celtis cf occidentalis, Taxus baccata, Acer negundo, Juglans regia.*  Shrub layer*: Crataegus monogyna, Cornus cf sanguinea, Philadelphus sp., Ligustrum vulgare.*  Herbaceous layer*: Plantago major, Poa trivialis, Taraxacum sp., Lolium perenne, Poa trivialis, Arctium sp., Ranunculus repens, Hedera helix, Rumex obtusifolius, Vinca minor.* | Tree layer*: Populus alba, Platanus x hispanica, Acer pseudoplatanus, Ginkgo biloba, Salix babylonica*, *Pinus nigra, Acer negundo, Typha latifolia.*  Herbaceous layer:  *Trifolium repens, Lolium perenne, Phragmites australis, Catalpa bignonioides, Parthenocissus quinquefolia, Dactylis glomerata, Silene latifolia subsp. alba, Sambucus ebulus, Daucus carota, Centaurea cf phrygia.* | Tree layer*: Platanus x hispanica*  Herbaceous layer*: Rumex sp*., *Capsella bursa-pastoris, Dactylis glomerata, Trifolium repens, Urtica dioica, Silene latifolia subsp. alba* |
| Type of soil | Clay | Clay | Clay |
| **Unmanaged urban areas** | **Băneasa** | **Pantelimon** | **Griviţa** |
| Geographical coordinates: | N: 44°29'36.8"  E: 26°04'11.9" | N: 44°26'00.3"  E: 26°12'33.9" | N: 44°29'40.6"  E: 26°03'09.7" |
| Altitude | 91 m | 66 m | 89 m |
| Slope | 5^0^ | 0 | 10^0^ |
| Exposure | North-East | - | North-East |
| Location within city | Periphery | Periphery | Periphery |
| Vegetation description (dominant plant species) | Tree layer*: Acer pseudoplatanus, Fraxinus excelsior, Populus x canadensis Populus nigra, Ulmus cf laevis, Prunus sp., Prunus cerasifera var. pissardii*  Shrub layer*: Rosa* sp., *Syringa vulgaris, Rhamnus cathartica, Crataegus monogyna*  Herbaceous layer*: Taraxacum sp.* , *Geum urbanum,* *Lolium perenne, Dactylis glomerata, Lepidium draba,* *Plantago major, Plantago lanceolata, Capsella bursa-pastoris* | Tree layer*: Acer negundo, Salix babylonica, Populus nigra “Italica”, Salix fragilis, Prunus cf spinosa, Pinus cf nigra, Populus cf x canadensis.*  Shrub layer*: Elaeagnus angustifolia, Crataegus monogyna*  Herbaceous layer*: Phragmites australis, Ranunculus sp., Bromus cf sterilis, Lepidium draba, Taraxacum sp., Geum urbanum, Arctium sp., Rumex cf acetosa, Parthenocissus quinquefolia, Chelidonium majus.* | Tree layer*: Prunus spinosa, Populus nigra “Italica”, Salix babylonica, Salix alba, Acer negundo.*  Shrub layer*: Sambucus ebulus, Crataegus monogyna, Rosa sp.*  Herbaceous layer*: Lepidium draba, Taraxacum sp., Phragmites australis, Poa annua, Trifolium repens*, *Phalaris arundinacea Carex riparia, Rumex cf obtusifolius, Urtica dioica, Lolium perenne.* |
| Type soil | Clay + sand+construction waste | Clay+ sand+construction waste | Clay+ sand+construction waste |
|  | **Lacul Morii** | **Fundeni** |  |
| Geographical coordinates: | N: 44^0^27’351”  E:026^0^01’002” | N: 44^0^27’497”  E: 026^0^09’056” |  |
| Altitude | 83 m | 64 m |  |
| Slope | 0 | 7^0^ |  |
| Exposure | - | North-West |  |
| Location within city | Periphery | City centre |  |
| Vegetation description (dominant plant species) | Tree layer*: Salix babylonica, Prunus sp.*  Herbaceous layer: *Capsella bursa-pastoris, Lolium perenne, Poa spp., Taraxacum sp., Lepidium draba, Heracleum sphondylium, Trifolium repens.* | Tree layer*: Salix alba, Salix cf fragilis, Salix babylonica, Prunus avium.*  Shrub layer*: Rosa sp.*  Herbaceous layer*: Galium aparine, Phragmites australis, Carex cf riparia, Lepidium draba.* |  |
| Type soil | Clay | Clay+ sand+construction waste |  |
