# Supplementary material for: Soil mite communities (Acari: Mesostigmata) as indicators of urban ecosystems in Bucharest, Romania
Source: Sci Rep. 2021 Feb 15;11:3794. doi: 10.1038/s41598-021-83417-4 (PMC7884781; doi:10.1038/s41598-021-83417-4)
Supplement: Supplementary file 3 — Supplementary Information 3. [file 41598_2021_83417_MOESM3_ESM.docx]

Soil mite communities (Acari: Mesostigmata) as indicators of urban ecosystems in Bucharest, Romania

Manu M.^1*#^, Băncilă R.I.^2,3#^, Bîrsan C.C.^1^, Mountford O.^4^, Onete M.^1^

^1^Romanian Academy, Institute of Biology Bucharest, Department of Ecology, Taxonomy and Nature Conservation, street Splaiul Independenţei, no. 296, zip code 0603100, PO-BOX 56-53, fax 040212219071, tel. 040212219202, Bucharest, Romania, email: minodoramanu@gmail.com; ciprian.birsan@ibiol.ro, marilena.onete@gmail.com

^2^Faculty of Natural Sciences, University Ovidius Constanţa, Constanţa, Romania

^3^Department of Biospeleology and Soil Edaphobiology, “Emil Racoviţă” Institute of Speleology, Romanian Academy, 13 Septembrie Road, No. 13, 050711, Bucharest, Romania, email: bancila_ralucaioana@yahoo.com

^4^Centre for Ecology and Hydrology, Maclean Building, Benson Lane, Crowmarsh Gifford, Wallingford, Oxfordshire, OX10 8BB, UK, email: om@ceh.ac.uk

Corresponding author email: minodoramanu@gmail.com

^#^ The authors had an equal contribution to the production of this article.

Appendix 3

Shepard plot, the scatter around the regression of the distances between each pair of communities against their observed dissimilarities, for non-metric multidimensional scaling analyses examining the differences across the management scenarios (a.) and different types of managed green areas (b.).


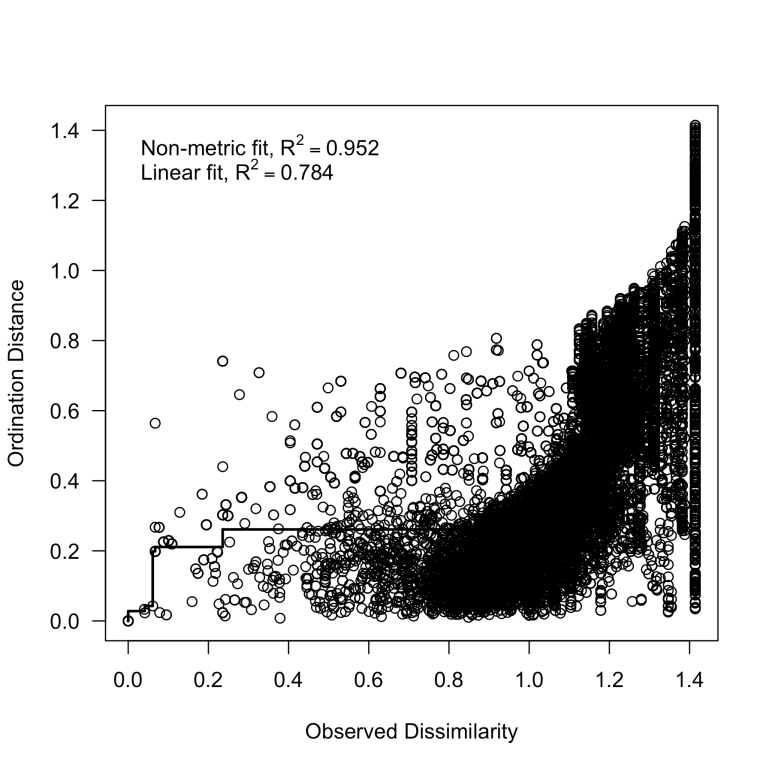


a.


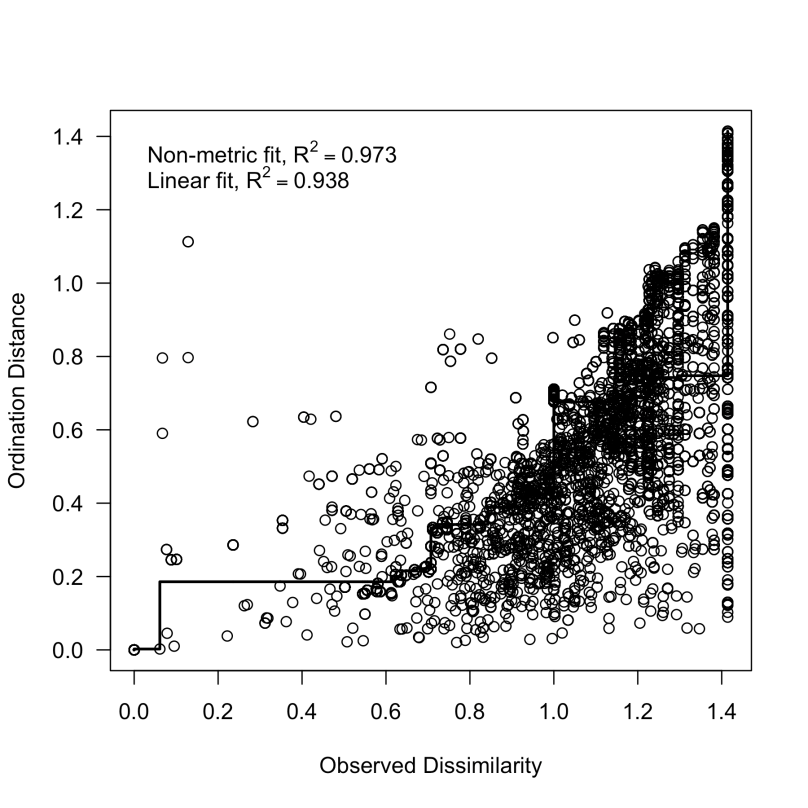


b.
