# Supplementary material for: Soil mite communities (Acari: Mesostigmata) as indicators of urban ecosystems in Bucharest, Romania
Source: Sci Rep. 2021 Feb 15;11:3794. doi: 10.1038/s41598-021-83417-4 (PMC7884781; doi:10.1038/s41598-021-83417-4)
Supplement: Supplementary file 4 — Supplementary Information 4. [file 41598_2021_83417_MOESM4_ESM.docx]

Soil mite communities (Acari: Mesostigmata) as indicators of urban ecosystems in Bucharest, Romania

Manu M.^1*#^, Băncilă R.I.^2,3#^, Bîrsan C.C.^1^, Mountford O.^4^, Onete M.^1^

^1^Romanian Academy, Institute of Biology Bucharest, Department of Ecology, Taxonomy and Nature Conservation, street Splaiul Independenţei, no. 296, zip code 0603100, PO-BOX 56-53, fax 040212219071, tel. 040212219202, Bucharest, Romania, email: minodoramanu@gmail.com; ciprian.birsan@ibiol.ro, marilena.onete@gmail.com

^2^Faculty of Natural Sciences, University Ovidius Constanţa, Constanţa, Romania

^3^Department of Biospeleology and Soil Edaphobiology, “Emil Racoviţă” Institute of Speleology, Romanian Academy, 13 Septembrie Road, No. 13, 050711, Bucharest, Romania, email: bancila_ralucaioana@yahoo.com

^4^Centre for Ecology and Hydrology, Maclean Building, Benson Lane, Crowmarsh Gifford, Wallingford, Oxfordshire, OX10 8BB, UK, email: om@ceh.ac.uk

Corresponding author email: minodoramanu@gmail.com

^#^ The authors had an equal contribution to the production of this article.

Appendix 4

Non-metric multidimensional scaling plot of samples compositions index for the 12 sites (Baneasa (B), Pantelimon (P), Grivita (G), Lacul Morii (LM), Fundeni (F), Carol (C), Plumbuita (PL), Tineretului (TN), Titan (TT), Floreasca (FL), Cringasi (CR) and management scenarios: protected areas (PA), managed green areas and (MGA) and unmanaged green areas (UGA) (a.) and different types of managed green areas: metropolitan parks (MtP), municipal parks (MnP) and district parks (DrP) (b.)


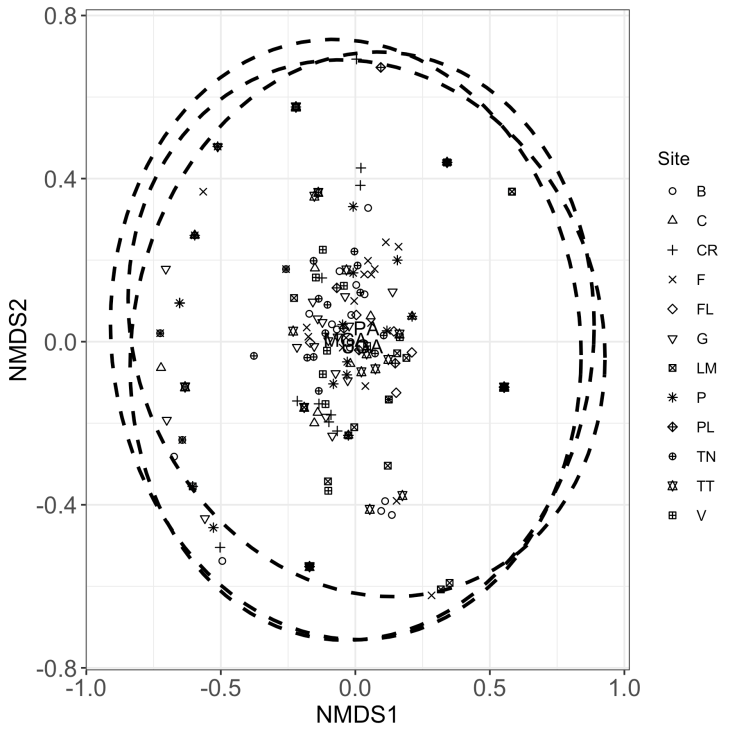


a.


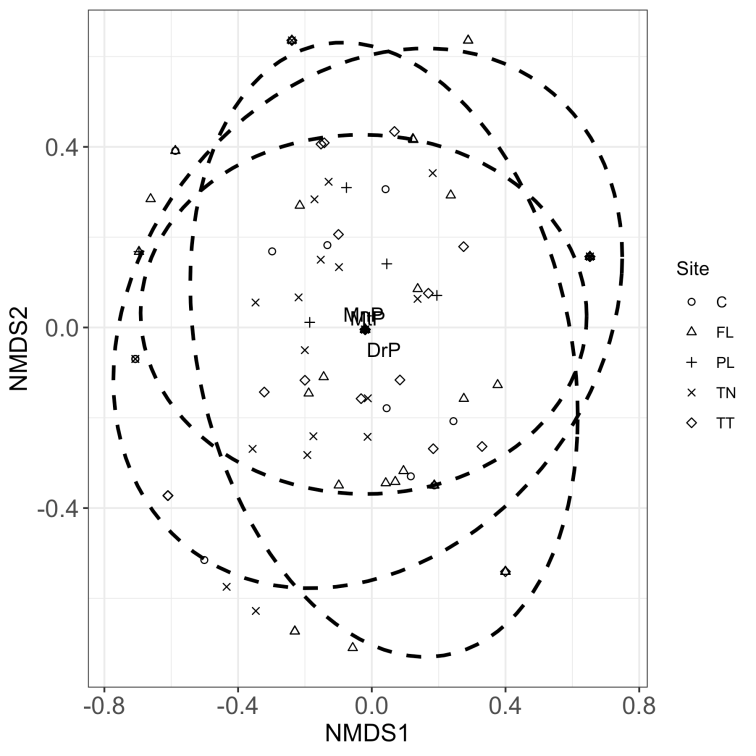


b.

.
