# Supplementary material for: Soil mite communities (Acari: Mesostigmata) as indicators of urban ecosystems in Bucharest, Romania
Source: Sci Rep. 2021 Feb 15;11:3794. doi: 10.1038/s41598-021-83417-4 (PMC7884781; doi:10.1038/s41598-021-83417-4)
Supplement: Supplementary file 5 — Supplementary Information 5. [file 41598_2021_83417_MOESM5_ESM.docx]

Soil mite communities (Acari: Mesostigmata) as indicators of urban ecosystems in Bucharest, Romania

Manu M.^1*#^, Băncilă R.I.^2,3#^, Bîrsan C.C.^1^, Mountford O.^4^, Onete M.^1^

^1^Romanian Academy, Institute of Biology Bucharest, Department of Ecology, Taxonomy and Nature Conservation, street Splaiul Independenţei, no. 296, zip code 0603100, PO-BOX 56-53, fax 040212219071, tel. 040212219202, Bucharest, Romania, email: minodoramanu@gmail.com; ciprian.birsan@ibiol.ro, marilena.onete@gmail.com

^2^Faculty of Natural Sciences, University Ovidius Constanţa, Constanţa, Romania

^3^Department of Biospeleology and Soil Edaphobiology, “Emil Racoviţă” Institute of Speleology, Romanian Academy, 13 Septembrie Road, No. 13, 050711, Bucharest, Romania, email: bancila_ralucaioana@yahoo.com

^4^Centre for Ecology and Hydrology, Maclean Building, Benson Lane, Crowmarsh Gifford, Wallingford, Oxfordshire, OX10 8BB, UK, email: om@ceh.ac.uk

Corresponding author email: minodoramanu@gmail.com

^#^ The authors had an equal contribution to the production of this article.

Appendix 5

Results of ANOVA like permutation test for canonical correspondence analysis to test the significance of each environmental variable prior to analysis.

|  | df | Chi Square | *F* | *P* |
| --- | --- | --- | --- | --- |
| T soil | 1 | 0.31 | 2.58 | 0.015 |
| RP | 1 | 0.25 | 2.06 | 0.005 |
| H soil | 1 | 0.23 | 1.9 | 0.01 |
| T air | 1 | 0.22 | 1.81 | 0.005 |
| H air | 1 | 0.18 | 1.52 | 0.01 |
| pH | 1 | 0.29 | 2.41 | 0.005 |
| Residual | 237 | 28.57 |  |  |
